# Loss of long noncoding RNA FOXF1-AS1 regulates epithelial-mesenchymal transition, stemness and metastasis of non-small cell lung cancer cells

**DOI:** 10.18632/oncotarget.11630

**Published:** 2016-08-26

**Authors:** Liyun Miao, Zhen Huang, Zhang Zengli, Hui Li, Qiufang Chen, Chenyun Yao, Hourong Cai, Yonglong Xiao, Hongping Xia, Yongsheng Wang

**Affiliations:** ^1^ Department of Respiratory Medicine, Nanjing Drum Tower Hospital Affiliated to Medical School of Nanjing University, Nanjing 210008, China; ^2^ Department of Pathology, Sir Run Run Hospital & Nanjing Medical University, Nanjing 211166, China; ^3^ Department of Laboratory Medicine, Longgang District Central Hospital, Longgang District, Shenzhen, Guangdong 518116, China; ^4^ Department of Respiratory Diseases, The Second Affiliated Hospital of Soochow University, Suzhou 215004, China; ^5^ Department of Clinical Pharmacy, School of Basic Medicine and Clinical Pharmacy, China Pharmaceutical University, Nanjing 210009, China; ^6^ Department of Radiation Oncology, The Affiliated Jiangsu Cancer Hospital, Nanjing Medical University, Nanjing 210009, China

**Keywords:** LncRNA, FOXF1-AS1, EMT, metastasis, lung cancer

## Abstract

Although recent evidence shows that long noncoding RNAs (lncRNAs) are involved in the regulation of gene expression and cancer progression, the understanding of the role of lncRNAs in lung cancer metastasis is still limited. To identify novel lncRNAs in non-small cell lung cancer (NSCLC), we profile NSCLC tumor and matched normal samples using GeneChip® Human Gene 2.0 ST Array, which provides the most accurate, sensitive, and comprehensive measurement of protein coding and lncRNA transcripts. We identified a panel of key factors dysregulated in lung cancer. Among them, the expression of FOXF1-AS1 was significantly downregulated in lung cancer. Stable overexpression of FOXF1-AS1 inhibits lung cancer cell migration and invasion by regulating EMT. Meanwhile, loss of FOXF1-AS1 mediates stem-like properties of lung cancer cells. Interestingly, we found that FOXF1-AS1 physically associates with PRC2 components EZH2 and loss of FOXF1-AS1 mediates cell migration and stem-like properties require EZH2. Loss of FOXF1-AS1 is also correlated with downregulation of FOXF1 in lung cancer. These results suggested that FOXF1-AS1 might regulate EMT, stemness and metastasis of NSCLC cells via EZH2, indicating it as a therapeutic target for future treatment of NSCLC.

## INTRODUCTION

As one of the most common causes of cancer related death of the world, lung cancer has become a severe public health problem [1]. Two main subtypes of lung cancer are named as non-small cell lung cancer (NSCLC) and small cell lung cancer, which accounts for approximately 80-85% and 15-20% respectively [2]. Although advances in the molecular carcinogenesis and new targeted therapies for NSCLC developed dramatically in the past few years [3–5], the overall survival of patients with this disease still remains low [6, 7]. The high mortality is probably related to early metastasis [8]; however, the mechanism underlying metastasis is still unknown yet.

Metastasis of NSCLC is a complex process and modulated by many steps [9]. NSCLC cells escape from the primary tumor to a new organ or tissue when metastasis begins. The main critical changes of progression and metastasis are epithelial-to-mesenchymal transition (EMT) and cancer stemness (CS) [10, 11], which play an important role in the embryonic development as well as the invasion and metastasis of cancer cells. Moreover, studies have demonstrated that the loss of epithelial adhesion and gain of mesenchymal features characterize EMT and CS [11]. To inhibit the process of metastasis and invasion of tumor cells seems vital to inhibit the tumor progression.

Long noncoding RNA (lncRNA) is consisted of more than 200 nucleotides in length. Increasing evidence has shown that lncRNAs trigger the initiation and progression of cancers [12]. Currently, a variety of lncRNAs including H19, HOTAIR, MALAT1, ANRIL and GAS5 have been identified to be tumor-associated especially in lung cancer [13–18]. However, more additional lung cancer-associated lncRNAs are still needed to be further investigated.

In this study, we profile NSCLC tumor and matched normal samples using GeneChip® Human Gene 2.0 ST Array, which provides the most accurate, sensitive, and comprehensive measurement of protein coding and lncRNA transcripts. We identified a panel of key factors dysregulated in lung cancer. Among them, the expression of FOXF1-AS1 was significantly downregulated in lung cancer. Loss of FOXF1-AS1 was also correlated with tumor migration and metastasis according to further investigation, which was then confirmed by overexpression experiments targeting FOXF1-AS1 in lung cancer cells to evaluate the changes in tumor cell behavior. Finally, we explained the function of EZH2 in the process of metastasis in the cells which were lack of FOXF1-AS1 expression. We also indicated that FOXF1 might be the target of FOXF1-AS1 in lung cancer cells. In summary, this study provided a novel insight on the role of FOXF1-AS1 in the migration, invasion and metastasis of lung cancer. Future studies should focus on discovering targeted therapies of lung cancer based on FOXF1-AS1.

## RESULTS

### LncRNA FOXF1-AS1 was lowly expressed in tissue samples from NSCLC patients

**Figure 1:**
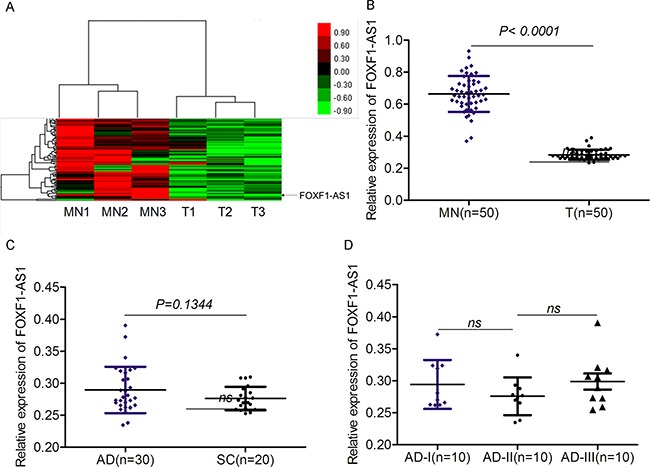
The expression of FOXF1-AS1 was significantly downregulated in lung cancer **A.** Hierarchical clustering showed the expression differences of lncRNAs between control matched normal samples (MN1, MN2, MN3) and tumor tissue samples (T1, T2, T3), of which the expression of LncRNA FOXF1-AS1 was significantly low in tumor tissue groups compared control groups. **B.** FOXF1-AS1 was lowly expressed in tumor tissues (n=50) than normal tissues (n=50) detected by real time qRT-PCR. p < 0.0001. **C.** The expression of FOXF1-AS1 mRNA had no significance between lung Adenocarcinoma (AD) (n=30) and Squamous Carcinoma (SC) (n=20) detected using real time qRT-PCR. P=0.1344. **D.** The expression of FOXF1-AS1 mRNA had no significance among ADs at different phases (n=10 for AD-I, AD-II and AD-III respectively).

To identify novel lncRNAs in non-small cell lung cancer (NSCLC), we profile NSCLC tumor and matched normal samples using GeneChip® Human Gene 2.0 ST Array, which provides the most accurate, sensitive, and comprehensive measurement of protein coding and lncRNA transcripts. We identified a panel of key factors dysregulated in lung cancer. Among them, the expression of FOXF1-AS1 was significantly downregulated in lung cancer (Figure 1A). The loss expression FOXF1-AS1 in lung cancer tissues was further validated by qRT-PCR (Figure 1B). Among the tumor tissues examined, there were 30 adeno-carcinomas (AD) and 20 squamous carcinomas (SC). Interestingly, the difference did not exist between these two types of lung cancers (Figure 1C) and even among different staging of AD as well (Figure 1D). Therefore, the expression of FOXF1-AS1 was significantly downregulated in non-small cell lung cancer.

### Stable overexpression of FOXF1-AS1 inhibits lung cancer cell migration and invasion

**Figure 2:**
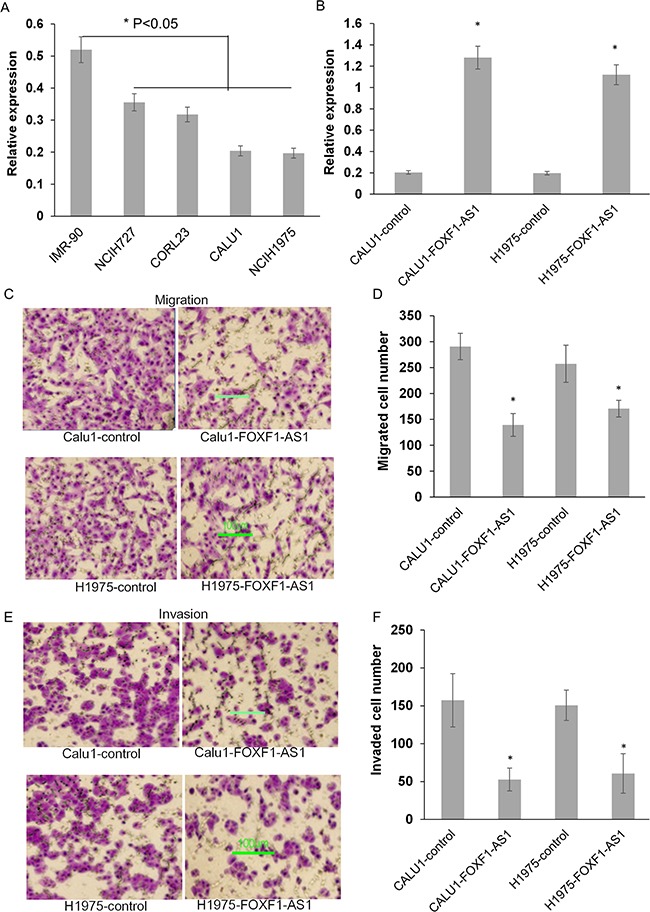
Stable overexpression of FOXF1-AS1 inhibited lung cancer cell migration and invasion **A.** The expression of FOXF1-AS1 was decreased variously among different lung cancer cell lines compared to normal IMR-90 cells. **B.** The expression of FOXF1-AS1 was assessed in CALU1 and NCIH1975 cells co-transfected with full length FOXF1-AS1 cDNA using plasmid vectors compared with control CALU1 and NCIH1975 cells. **C.** The representative images showed the effect of FOXF1-AS1 overexpression on migration of CALU1 and NCIH1975 cells by transwell migration assay. **D.** The number of migration cells was quantified among three different wells. **E.** The representative images showed the effect of FOXF1-AS1 overexpression on invasion of CALU1 and NCIH1975 cells by transwell matrigel invasion assay. **F.** The number of invasive cells was quantified among three different wells.

The low expression of FOXF1-AS1 was also observed in various types of lung cancer cells, especially CALU1 and NCIH1975, compared to the normal human lung diploid fibroblast cell IMR-90 (Figure 2A). To investigate the critical role of FOXF1-AS1 in lung cancer, full length FOXF1-AS1 cDNA was transfected into lung cancer cells by plasmid vectors. The expression of FOXF1-AS1 was then confirmed by qRT-PCR in CALU1-FOXF1-AS1, CALU1-control, H1975- FOXF1-AS1 and H1975-control cells (Figure 2B).

To investigate the function of FOXF1-AS1 on CALU1 and NCI-H1975 cell migrant, CALU1-FOXF1-AS1 cell and H1975-FOXF1-AS1 cell that overexpressed FOXF1-AS1 or their negative control were allowed to migrate through a transwell membrane into complete media. Compared to the negative control, overexpression of FOXF1-AS1 inhibits the motility of both two of the transfected cells (Figure 2C). The number of migrant cells was shown in Figure 2D. Then, to access cell invasion capability, CALU1-FOXF1-AS1 cell and H1975- FOXF1-AS1 cell were plated on membranes pre-coated with matrigel. As shown in Figure 2E and 2F, FOXF1-AS1 overexpression significantly inhibits the invasion of the two transfected cells.

### FOXF1-AS1 regulates epithelial-mesenchymal transition in lung cancer cells

**Figure 3:**
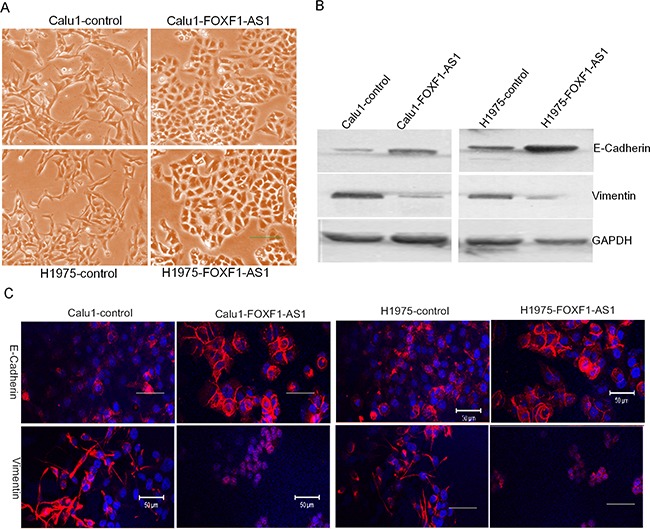
FOXF1-AS1 regulated epithelial-mesenchymal transition in lung cancer cells **A.** The representative image of CALU1 and NCIH1975 cells and the stable transfected with full length FOXF1-AS1 cDNA through light microscope. The morphological changes of CALU1 and NCIH1975 cells from a fibroblastoid appearance elongated spindle shape to cobblestone shape was observed after stable overexpression FOXF1-AS1, which is like mesenchymal to epithelial transition. **B.** Western blot analysis of EMT markers E-Cadherin and Vimentin after transfection. Note the decreased Vimentin and increased E-Cadherin in both CALU1 and NCIH1975 cells transfected with full length FOXF1-AS1 cDNA. The expression of GAPDH was used as an internal control. **C.** The representative images of IF staining for E-Cadherin and Vimentin. Each protein was detected using specific antibodies. The changes in the signal of E-Cadherin and in the signal of Vimentin were clearly detected in the cells.

By light microscope, we observed the morphological changes of CALU1 and NCIH1975 cells from a fibroblastoid appearance elongated spindle shape to cobblestone shape after stable overexpression FOXF1-AS1 (Figure 3A), which is like mesenchymal to epithelial transition. So the two proteins which were related to metastasis E-Cadherin and Vimentin were detected by western blot, indicating that the overexpression of FOXF1-AS1 decreased Vimentin and increased E-Cadherin in both CALU1 and NCIH1975 cells (Figure 3B). The result was also confirmed using immunofluorescence (IF) analysis with a confocal laser-scanning microscope (Figure 3C).

### Loss of FOXF1-AS1 mediates stem-like properties of lung cancer cells

**Figure 4:**
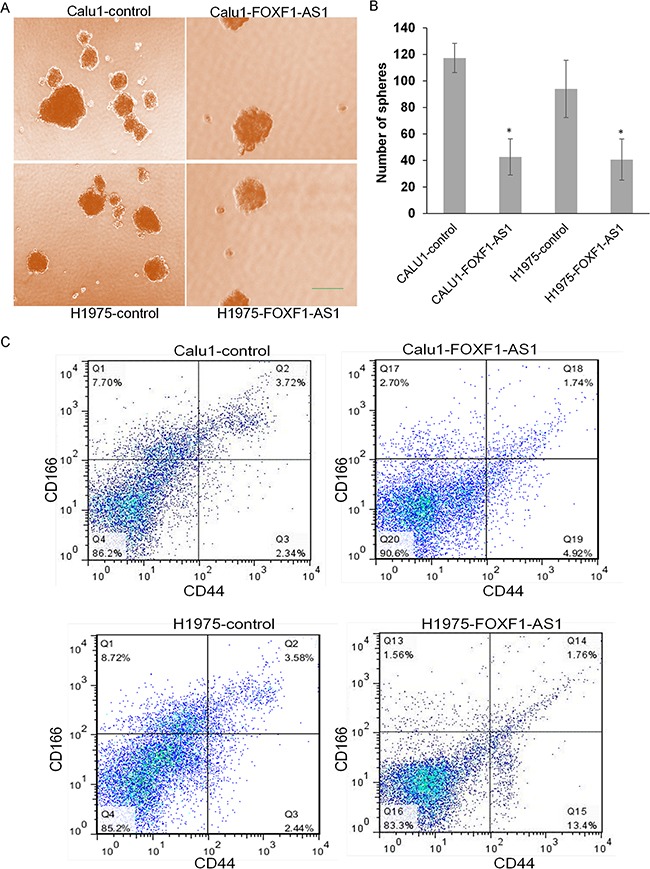
Loss of FOXF1-AS1 mediates stem-like properties of lung cancer cells **A.** The representative images of stem-like spheres in CALU1, NCIH1975 and their two transfected ones using light microscope. **B.** Numbers of the stem-like spheres in each group. The bars were calculated as the total of three wells from each group photographed by light microscope. The overexpression of FOXF1-AS1 inhibited sphere formation of lung cancer cells to stem-like cells significantly. *p<0.05. **C.** The expression of CD166 and CD44 in the cells of CALU1, NCIH1975, CALU1-FOXF1-AS1 and NCIH1975-FOXF1-AS1 detected by flow cytometry analysis. Stem-like lung cancer cells tended to express CD166 and CD44. The overexpression of FOXF1-AS1 downregulates stem-like cell population in CALU1 and NCIH1975 cells.

Considering stem-like cells as an important factor for tumor cell EMT and metastasis, we also examined the sphere formation ability of stem-like cells in high expression and low expression groups of both CALU1 and NCIH1975 cells. The result showed that overexpression of FOXF1-AS1 significantly inhibits the sphere formation ability of CALU1 and NCIH1975 cells (Figure 4A and 4B). Using flow cytometry analysis, we could get the ratio of stem-like cells (CD166+CD44+ population) decreased from 3.72% in CALU1 to 1.74% in CALU1- FOXF1-AS1 and similarly from 3.58% in NCIH1975 to 1.76% in NCIH1975- FOXF1-AS1. The difference between pre-transfected cells and post-transfected cells was significant so as to demonstrate that the overexpression of FOXF1-AS1 was able to suppress stem-like change of lung cancer cells (Figure 4C).

### FOXF1-AS1 physically associates with PRC2 components EZH2 and loss of FOXF1-AS1 mediates cell migration and stem-like properties require EZH2

**Figure 5:**
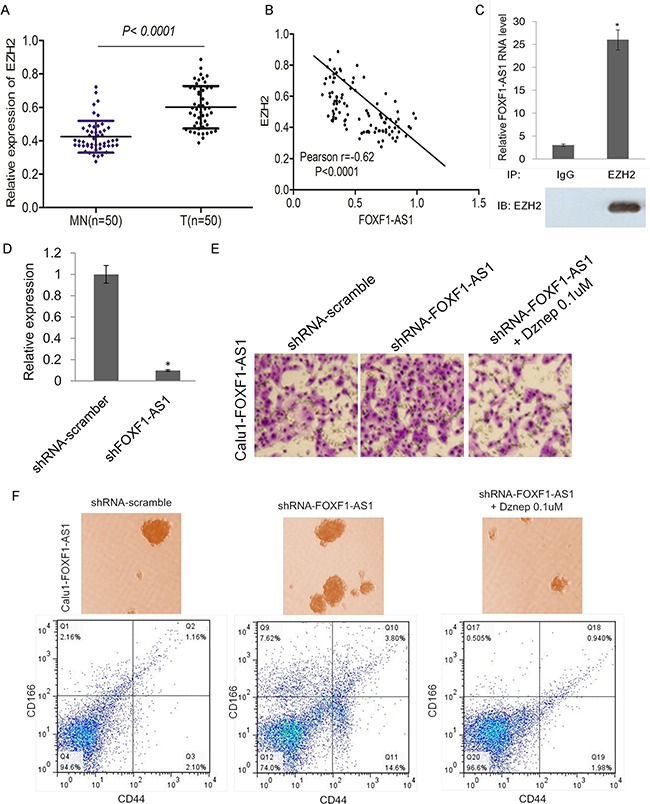
FOXF1-AS1 physically associates with PRC2 components EZH2 and loss of FOXF1-AS1 mediates cell migration and stem-like properties require EZH2 **A.** mRNA of EZH2 was highly expressed in tumor tissues (n=50) than normal tissues (n=50) detected by real time-qPCR. p < 0.0001. **B.** The expression of FOXF1-AS1 and EZH2 was inversely correlated with each other in lung cancer tissues. Pearson r=−0.62. p<0.0001. **C.** Binding of FOXF1-AS1 to EZH2 complex in CALU1 cells, shown by RNA immunoprecipitation followed qRT-PCR (mean±SD, n=3, *p < 0.05 versus lgG). **D.** Relative FOXF1-AS1 expression was analyzed by qRT-PCR after CALU1-FOXF1-AS1 cell transduction with shFOXF1-AS1 transfection and was represented on the bar graphs. Data showed that the expression of FOXF1-AS1 was suppressed significantly.*p<0.05. **E.** The effect of shFOXF1-AS1 and co-treatment with EZH2 inhibitor Dznep on migration of CALU1-FOXF1-AS1 cells was detected by transwell assay. Blocking the function of EZH2, shFOXF1-AS1 could not induce the migration of CALU1-FOXF1-AS1 cells. **F.** The effect of shFOXF1-AS1 and co-treatment with EZH2 inhibitor Dznep on stem-like sphere formation and CD166+CD44+ cell population of CALU1-FOXF1-AS1 cells was detected by light microscope and flow cytometry analysis. Blocking the function of EZH2, shFOXF1-AS1 could not promotes stem-like sphere formation and CD166+CD44+ cell population of CALU1-FOXF1-AS1 cells.

To explore whether EZH2 was related to the function of LncRNA FOXF1-AS1, we analyzed the expression of EZH2 in lung cancer tissues compared with normal tissues, showing that EZH2 expressed highly in tumor tissues (Figure 5A). The expression of FOXF1-AS1 and EZH2 was inversely correlated with each other (Figure 5B). Support Vector Machine (SVM) was used in RNA-Protein Interaction Prediction (RPISeq) for the prediction of lncRNA–protein interactions, which indicated that FOXF1-AS1 probably interacts with EZH2. Next we sought to determine whether FOXF1-AS1 regulates CALU1 cell invasion via EZH2. An RNA immunoprecipitation assay of EZH2 showed that FOXF1-AS1could bind to EZH2 (Figure 5C). Thus, we could have the conclusion that FOXF1-AS1 interacted with EZH2 to modulate the metastasis of lung cancer cells.

To further confirmation of the results, CALU1-FOXF1-AS1 cell line which expressed highly of FOXF1-AS1 was transfected with shFOXF1-AS1, leading to a significant suppression of FOXF1-AS1 expression (Figure 5D). The tranwell migration assay result showed that knockdown of FOXF1-AS1 significantly promotes CALU1-FOXF1-AS1 cell migration, while blocking EZH2 by DZnep overcome the effect of FOXF1-AS1 knockdown (Figure 5E). The sphere formation and flow cytometry analysis also indicated that knockdown of FOXF1-AS1 significantly promotes the stem-like sphere formation ability and CD166+CD44+ cell population of CALU1-FOXF1-AS1 (Figure 5F). Together, these results suggested that FOXF1-AS1 physically associates with PRC2 components EZH2 and loss of FOXF1-AS1 mediates cell migration and stem-like properties require EZH2.

### Loss of FOXF1-AS1 is correlated with downregulation of FOXF1 in lung cancer

**Figure 6:**
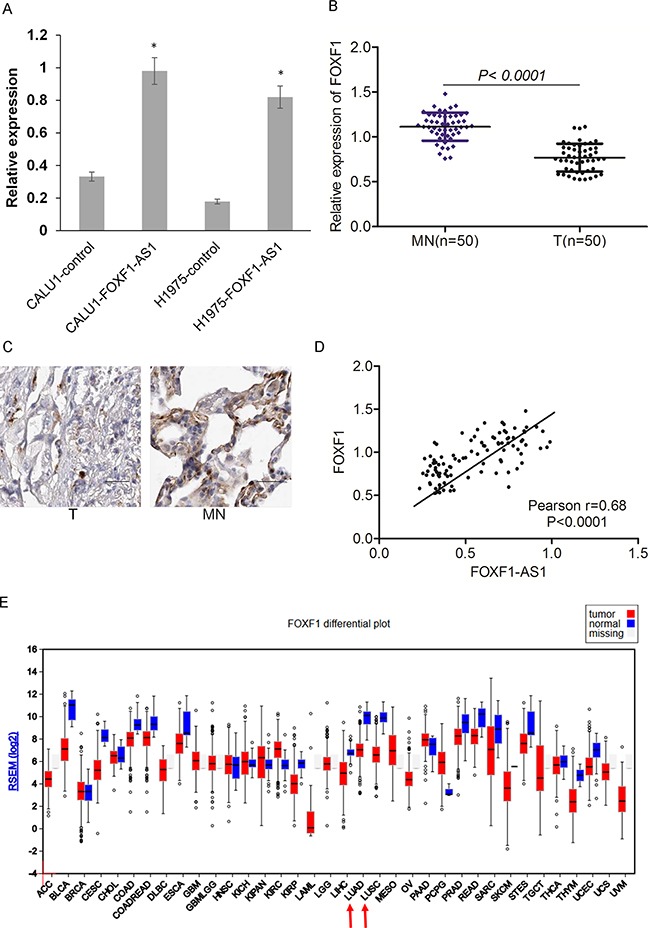
Loss of FOXF1-AS1 is correlated with downregulation of FOXF1 in lung cancer **A.** The expression of FOXF1 was assessed in CALU1 and NCIH1975 cells co-transfected with full length FOXF1-AS1 cDNA using plasmid vectors compared with control CALU1 and NCIH1975 cells by real time-qPCR. **B.** mRNA of FOXF1 was lowly expressed in tumor tissues (n=50) than normal tissues (n=50) detected by real time-qPCR. p < 0.0001. **C.** The expression of FOXF1 protein level was lowly expressed in tumor tissues than normal tissues detected by IHC. **D.** The expression of FOXF1-AS1 and FOXF1 was positive correlated with each other in lung cancer tissues. Pearson r=0.68. p<0.0001. **E.** the significantly low expression of FOXF1 in lung adeno-carcinomas (LUAD) and lung squamous carcinomas (LUSC) was also observed in The Cancer Genome Atlas (TCGA).

To explore whether FOXF1 was the target gene of LncRNA FOXF1-AS1, we detected the expression of FOXF1 in the cells of CALU1, CALU1-FOXF1-AS1, NCIH1975 and NCIH1975- FOXF1-AS1 by qRT-PCR. The results showed that FOXF1 had a high expression in the cells which highly expressed LncRNA FOXF1-AS1 (Figure 6A). The expression of FOXF1 and its protein level was also evaluated in the tumor tissues and normal tissues, indicating that FOXF1 had a low expression in tumor tissues which were loss of FOXF1-AS1 (Figure 6B and 6C). Consistently, the significantly low expression of FOXF1 in lung adeno-carcinomas (LUAD) and lung squamous carcinomas (LUSC) was also observed in The Cancer Genome Atlas (TCGA) (Figure 6D).

## DISCUSSION

Accumulating evidence has indicated that lncRNAs play an important role in the cancer pathogenesis. The function of lncRNAs in lung cancer, especially NSCLC, has been explored for many years using microarray tiling array methods [19]. Notably, among the differentially expressed lncRNAs which have already been observed, associations between HOX antisense intergenic RNA (HOTAIR), metastasis-associated- in-lung-adenocarcinoma-transcript-1 (MALAT1), BRAF activated noncoding RNA (BANCR),SPRY4 intronic transcript 1 (SPRY4-IT1) and antisense noncoding RNA in the INK4 locus (ANRIL) with NSCLC have been reported, which directs that these lncRNAs may be the novel modulators in the process of metastasis in human NSCLC [14, 20–23]. However, whether other lncRNAs are also involved in this process still remains largely uncharacterized.

Currently, no study has identified FOXF1-AS1 as a metastasis regulator that is important for the tumor aggression. In this study, we originally detect the low expression of FOXF1-AS1 in NSCLC tissues by microarray method. Previous studies suggested that migration, invasion and metastasis were the key factors for therapeutic failure of NSCLC. Here, we firstly identify the relationship between loss of FOXF1-AS1 and EMT in lung cancer. EMT is a critical step in metastasis; E-cadherin and vimentin are two well-established hallmark of EMT. Therefore, we evaluated the expression of these two proteins in vitro to confirm that loss of FOXF1-AS1 expression induced the EMT of lung cancer cells, leading the tumor cells more aggressive. Furthermore, loss of FOXF1-AS1 was also associated with stemness of tumor cells, which had an obvious effect on cell metastasis as well. In addition, although the essential role of EZH2 in the process of metastasis has been reported with other lncRNA [24], its effect with FOXF1-AS1function was confirmed by us for the first time. The target of FOXF1-AS1 might be FOXF1, which was also investigated in this study.

In conclusion, we found that FOXF1-AS1 was significantly downregulated lncRNA in NSCLC. Loss of FOXF1-AS1 expression was associated with tumor migration, invasion and metastasis, suggesting that FOXF1-AS1 may be a useful targeted biomarker for the therapy of NSCLC. We also found that that the function of FOXF1-AS1 was related to EZH2 and it regulated the tumor biological behavior by targeting FOXF1, indicating that further investigation of FOXF1-AS1 might lead to the development of novel NSCLC therapies.

## MATERIALS AND METHODS

### Cell culture

The NSCLC cell lines were obtained from American type culture collection (ATCC). The cell lines were cultured in RPMI 1640 (Gibco, Grand Island, NY, USA) with 10 % fetal bovine serum (FBS) (HyClone, Camarillo, CA, USA) as well as 100 U/ml penicillin and 100 μg/ml streptomycin (Invitrogen, Carlsbad, CA, USA). Cells were maintained in a humidified incubator in the presence of 5 % CO2 at 37 °C. All cell lines have been passaged for fewer than 6 months.

### RNA extraction and GeneChip® Human Gene 2.0 ST Array

Total RNA was extracted from FFPE using RNAprep Pure FFPE Kit (TIANGEN Biotech, Beijing, China) following the manufacturer's instruction. Total RNA from each sample was quantified. The preparation of samples for microarray hybridization was performed based on the manufacturer's protocols. Briefly, cDNA was regenerated through a random-primed reverse transcription (RT) using a dNTP mix containing dUTP. After cDNA was hybridized to the arrays at 60 rpm for 18h at 45°C, the chips were processed in a Genechip Fluidics Station 450 (Affymetrix). Microarray images were collected by Scanner 3000 7G (Affymetrix), and data were extracted using Affymetrix GCOS software (Affymetrix). The scanned probe array data were normalized using robust multi-array average. Differentially expressed probe sets were selected by one-way analysis of variance (ANOVA).

### Real-time quantitative RT-PCR

Total RNA from cells was extracted using TRIzol Reagent (Invitrogen, Carlsbad, CA, USA). After total RNA was extracted according to the method mentioned above. cDNA was synthesized using the Primer-Script™ One Step RT-PCR Kit (TaKaRa, Dalian, China). The cDNA template was amplified by real time-qPCR using the SYBR® Premix Dimmer Eraser kit (TaKaRa, Dalian, China) carried out on ABI 7500 system (Applied Biosystems, CA, USA) using a SYBR Premix Ex Taq II kit (TaKaRa) according to the manufacturer's instructions. GAPDH was measured as an internal control, and mRNA values were normalized to GAPDH. The relative expression fold change of mRNAs was calculated by the 2 ^−ΔΔCt^method.

### Transwell migration and invasion assay

Transwell migration and invasion assay were performed using transwell chambers without (migration) or with (invasion) Matrigel (BD Sciences, Sparks, MD, USA). The CALU1 and H1975 cells were seeding in the six well plates and tranfected with pcDNA3.1- FOXF1-AS1 or pcDNA3.1 control plasmid using Lipofectamine 2000 (Invitrogen, Carlsbad, CA, USA) according to the manufacturer's instructions. The stable clones were selected by adding G418 and the expression of FOXF1 was valided by qRT-PCR. The stable transfected CALU1-FOXF1-AS1 and H1975- FOXF1-AS1 and control cells at an approximate density of 1 × 10^5^ were suspended and seeded in the upper chambers of 24 well plates with FBS free medium. After 18 h, cells that migrated were stained by 0.5 % crystal violet solution for 15 min and counted. For invasion assays, transwell membrane was prepared with matrigel for plating infected cells. After 24 h, cells that migrated were stained by 0.5 % crystal violet solution for 15 min and counted.

### Western blot analysis

The stable transfected CALU1-FOXF1-AS1 and H1975- FOXF1-AS1 and control cells were prepared for western blot as previously described [25]. The antibody used included E-cadherin, Vimentin antibody (Cell signaling, USA), GAPDH, FOXF1 and EZH2 antibody produced in rabbit (Abcam).Each of the ratio was determined to the values obtained for GAPDH.

### Immunofluorescence (IF) analysis

The stable transfected CALU1-FOXF1-AS1 and H1975- FOXF1-AS1 and control cells were seeded in the BD Falcon™ 8-well CultureSlide and incubated with primary antibodies against E-cadherin, or Vimentin and then incubated with Alexa Fluor® 594 Goat Anti-Rabbit IgG (Invitrogen). The culture slides were counterstained with Hoechst 33342 and imaged with a confocal laser-scanning microscope (Carl Zeiss). Data were processed with Adobe Photoshop 7.0 software for analysis.

### Sphere formation

Sphere culture was performed as previously described [26]. Briefly, stable transfected CALU1-FOXF1-AS1 and H1975- FOXF1-AS1 and control cells were plated in triplicate in ultra-low attachment plates in serum-free DMEM/F12 medium supplemented supplemented with 5 μg/mL insulin (Sigma), 20 ng/mL human recombinant epidermal growth factor (EGF, Peprotech) and 10 ng/mL basic fibroblastic growth factor (bFGF, Peprotech). Cells were plated at the indicated density and from 1,000–10,000 cells/ml in subsequent passage. Sphere formation was assessed 2 weeks after seeding the cells.

### Flow cytometric assays

The stable transfected CALU1-FOXF1-AS1 and H1975- FOXF1-AS1 and control cell suspensions were counted and incubated with primary antibodies CD44 and CD166 (Sigma). Cells were incubated for 45 min. Gating was established using Propidium Iodide (PI)-exclusion for viability. Flow cytometry analysis was performed with ≥1×10^5^ cells using the BD FACSCanto II (Becton Dickinson, San Diego, USA).

### RNA immunoprecipitation

RNA immunoprecipitation was performed using Magna RIP RNA-Binding Protein Immunoprecipitation Kit (17-700, Millipore) according to manufacturer's instructions. Binding of FOXF1-AS1 to EZH2 complex in CALU1 cells, shown by RNA immunoprecipitation followed qRT-PCR to detect the relative FOXF1-AS1 RNA level.

### Immunohistochemistry

The avidin-biotin-peroxidase complex (ABC) method was used to do the immunohistochemistry procedure. The antibody used in the experiment included goat anti-FOXF1 polyclonal antibody (Santa Cruz Biotechnology, USA), rabbit-anti-goat IgG (Wuhan Boster Biological Technology, China). Images of tissue sections for the FOXF1 immunohistochemistry procedure were acquired using an OLYMPUS BX43 microscope (Olympus Corporation, Tokyo, Japan). Negative control tests were conducted via primary antisera pre-absorption using its respective antigen.
